# Characteristics and Clinical Outcomes of Lyme Arthritis: A Retrospective Study

**DOI:** 10.1093/ofid/ofag368

**Published:** 2026-06-19

**Authors:** Khalid Abu-Zeinah, Sofia Molina Garcia, Madiha Fida, Omar Abu Saleh

**Affiliations:** Department of Internal Medicine, Mayo Clinic, Rochester, Minnesota, USA; Division of Public Health, Infectious Diseases and Occupational Medicine, Mayo Clinic, Rochester, Minnesota, USA; Division of Public Health, Infectious Diseases and Occupational Medicine, Mayo Clinic, Rochester, Minnesota, USA; Division of Public Health, Infectious Diseases and Occupational Medicine, Mayo Clinic, Rochester, Minnesota, USA

**Keywords:** antibiotic treatment, *Borrelia burgdorferi*, Lyme arthritis, polymerase chain reaction, postantibiotic Lyme arthritis

## Abstract

**Background:**

Lyme arthritis is the most common late manifestation of disseminated Lyme disease in the United States. While most patients respond to antibiotic therapy, a subset develops postantibiotic Lyme arthritis (PALA), characterized by persistent noninfectious inflammatory arthritis. Data describing predictors of PALA and treatment patterns remain limited.

**Methods:**

We conducted a retrospective observational cohort study of patients with polymerase chain reaction (PCR)−confirmed native joint Lyme arthritis treated at our institution from 2004 to 2025. Clinical, laboratory, imaging, treatment, and outcome data were abstracted from electronic health records. Continuous variables were compared using the Mann–Whitney *U* test, and categorical variables using Fisher's exact test.

**Results:**

Seventy-seven patients with PCR-confirmed native joint Lyme arthritis were identified: 64 adults and 13 children. Children more frequently presented with fever and had significantly higher synovial fluid white blood cell counts compared with adults. Knee monoarthritis was the predominant presentation in both groups. Among adults, 41 (64%) achieved complete symptomatic resolution following antibiotic therapy, while 19 (29.7%) developed PALA. Adults who developed PALA had significantly lower synovial fluid white blood cell counts and more extensive joint involvement. The use of intravenous antibiotics and longer total antibiotic duration was more common among PALA patients without evidence of improved outcomes.

**Conclusions:**

In this cohort of PCR-confirmed native joint Lyme arthritis, a substantial proportion of adults developed PALA. Failure to respond to initial antibiotic therapy and certain clinical features may identify patients at increased risk. These findings support judicious antibiotic use and early consideration of noninfectious mechanisms in persistent arthritis.

## Teaser

In our cohort of PCR-confirmed native joint Lyme arthritis, nearly one-third of adults developed postantibiotic Lyme arthritis (PALA). Prolonged or intravenous antibiotic therapy was frequently used among patients who progressed to PALA, without evidence of improved outcomes.

Lyme disease, caused by spirochetes in the *Borrelia burgdorferi* sensu lato complex, is the most common vector-borne infection in the United States [1]. The incidence of Lyme disease has risen over the past several decades [1]. The infection is primarily transmitted to humans by *Ixodes* ticks, which are prevalent in the Northeastern, Mid-Atlantic, and Midwestern regions of the United States but are also found elsewhere across the globe [2, 3]. Most cases of Lyme disease initially present with a characteristic skin rash, erythema migrans (EM), and are treated promptly. However, in some individuals, the rash may not occur, or the tick bite or rash may go unnoticed, resulting in dissemination of infection [4].

In the United States, Lyme arthritis is the most common late manifestation of disseminated Lyme disease. In contrast, disseminated Lyme in Europe more frequently involves neurological and dermatological complications [5, 6]. This difference has been largely attributed to regional variations in *Borrelia* species, which can differ in their disease course: *B burgdorferi* sensu stricto predominates in the United States, whereas *B afzelii* and *B garinii* are more common in Europe [5, 6].

Lyme arthritis usually presents as monoarticular or oligoarticular inflammatory arthritis, commonly affecting large joints, particularly the knee [2, 4]. Symptom onset is usually delayed, occurring months after initial infection. Reported timeframes of symptom onset range from 4 days to 2 years, with a mean of approximately 6 months [7]. Diagnosing Lyme arthritis can be challenging, as culturing *Borrelia* species from synovial fluid is difficult. Accordingly, serum 2-tier serological testing remains the mainstay diagnostic tool. As Lyme arthritis is often a late manifestation of Lyme disease, IgG positivity is expected [2]. Another diagnostic tool is synovial fluid polymerase chain reaction (PCR) testing for *B burgdorferi* DNA, which provides diagnostic specificity but has variable reported sensitivity (40%–96%) [2]. Current guidelines do not require PCR testing for Lyme arthritis diagnosis, which is based on compatible clinical features and serologic testing [8]. However, synovial fluid PCR testing may be useful in situations of diagnostic uncertainty, particularly among seropositive patients with treated prior Lyme disease or those living in endemic areas, as seroreactivity following treated infection may persist for years, reducing the diagnostic specificity of serologic testing alone [8].

The Infectious Diseases Society of America (IDSA) guidelines recommend an initial 4-week course of oral antibiotics (doxycycline or amoxicillin) for Lyme arthritis. If symptoms persist, a second 4-week course of oral antibiotics or a 2–4 week course of intravenous antibiotics (ceftriaxone) may be administered [8]. Most patients respond to antibiotic treatment; however, a subset (reported rates ranging from approximately 10%–23%) develop persistent synovitis and arthritis despite appropriate therapy [2, 8, 9]. This condition, known as postantibiotic Lyme arthritis (PALA), is believed to result not from persistent infection, but from a dysregulated proinflammatory response with an autoimmune component [10]. The risk factors predisposing to PALA development are not fully understood. Host genetic factors may play a role, with specific HLA-DR alleles associated with an increased risk [10]. Additionally, particular strains of *B burgdorferi*, notably the OspC type A (RST1) strain, have been linked to a higher likelihood of progressing to PALA [10]. Some studies have suggested that intra-articular corticosteroid injections administered prior to antibiotic treatment can be associated with the development of PALA, but findings have been inconsistent [6, 9].

The aim of this study was to characterize demographics, clinical features, and long-term outcomes of patients with Lyme arthritis and identify factors associated with PALA development. We also aim to evaluate antibiotic usage patterns in Lyme arthritis treatment.

## METHODS

We conducted a retrospective observational cohort study of patients diagnosed with Lyme arthritis at Mayo Clinic in Rochester, Minnesota, and affiliated Mayo Clinic Health System sites in Minnesota and Wisconsin. These institutions serve a predominantly Midwestern US population in a Lyme-endemic region. This study was approved by the Mayo Clinic Institutional Review Board (IRB #25-001994), with waivers of informed consent and assent due to the minimal-risk, retrospective nature of the research.

We identified patients with Lyme arthritis based on microbiology laboratory records from our institution between January 1, 2004, and February 28, 2025. Eligible patients had ≥1 positive result for *B burgdorferi* DNA by polymerase chain reaction (PCR) in synovial fluid. To maximize diagnostic specificity and reduce misclassification bias, only patients with PCR-confirmed native joint Lyme arthritis were included in our study. Although serologic testing is more sensitive than PCR and is expected to be positive in nearly all patients with Lyme arthritis, seropositivity alone may be less specific in endemic regions where prior exposure is common. A highly specific case definition was therefore chosen to ensure that patients included had definite Lyme arthritis. Descriptive analyses of patients with ICD-coded Lyme arthritis and positive serology during the same period were also performed (Supplementary Appendix).

PCR testing was performed in our institution's microbiology laboratory using a validated, laboratory-developed real-time PCR assay targeting the *plasminogen-binding protein (oppA2)* gene of the *B burgdorferi* sensu lato complex [11]. Nucleic acids extracted from clinical specimens were amplified using fluorescence resonance energy transfer hybridization probes. The assay detects and differentiates *B burgdorferi sensu stricto*, *B mayonii*, *B afzelii*, and *B garinii* based on melting-curve analysis.

Lyme disease serologic testing evolved at our institution's laboratory over the study period. Prior to October 2021, testing was performed using the standard 2-tier approach, with an initial enzyme immunoassay followed by confirmatory IgM and IgG immunoblot. Thereafter, a modified 2-tier approach was used, in which positive or equivocal enzyme immunoassays are followed by reflex IgM and IgG enzyme immunoassays.

Patient data were abstracted from electronic health records using a standardized form and entered into the Research Electronic Data Capture (REDCap) system by one investigator. Collected variables included demographics, clinical characteristics (symptom duration, joint involvement, tick exposure, fever, and pretreatment intra-articular corticosteroid use), laboratory values (erythrocyte sedimentation rate [ESR], C-reactive protein [CRP], synovial fluid analysis, serology), imaging findings, treatment, and clinical outcomes (response to therapy and need for additional treatment).

Clinical response to treatment was defined as resolution of joint symptoms and inflammation based on physician documentation at follow-up encounters, incorporating patient-reported symptoms and objective clinical assessment of synovitis. Microbiological response was defined as a negative synovial fluid PCR result for *B burgdorferi*, regardless of subsequent treatment. PALA was defined as persistent inflammatory arthritis, characterized by objective synovitis on clinical examination (eg, joint effusion, swelling, warmth, or restricted range of motion), despite receiving at least 2 courses of antibiotic therapy (oral or intravenous), or in the setting of microbiological clearance on repeat synovial fluid PCR testing. Persistent synovitis was determined based on documentation at follow-up appointments occurring within 90 days after completion of a course of antibiotic therapy. Laboratory markers of inflammation and repeat synovial fluid analyses were not required for PALA classification given that they were inconsistently performed. An antibiotic course was defined as either 4 weeks of appropriate oral antibiotics or 2–4 weeks of appropriate intravenous antibiotics. One adult patient received 14 days of oral doxycycline immediately followed by intravenous ceftriaxone. Because the duration of oral therapy was insufficient to constitute a complete oral course and escalation occurred without an intervening treatment interruption, this treatment sequence was classified as a single course of antibiotic therapy.

Descriptive statistics were used to summarize demographic and clinical characteristics. Continuous variables were reported as medians with interquartile ranges. Categorical variables were expressed as frequencies and percentages. Comparisons between groups were made using the Mann–Whitney *U* test for continuous variables and the Fisher's exact test for categorical variables. Analyses were conducted using RStudio version 4.2.2 (R Foundation for Statistical Computing, Vienna, Austria).

## RESULTS

### Characteristics of Lyme Arthritis

Seventy-seven patients were identified with PCR-confirmed *B burgdorferi* in native joint synovial fluid. Of these, 83.1% (64/77) were adults (≥18 years), and 16.9% (13/77) were children (<18 years). Table 1 outlines demographic and clinical characteristics of the patient cohort, stratified by age group. There was a male predominance in both adults (81.3% [52/64]) and children (61.5% [8/13]). Known tick exposure was reported in 23.4% (15/64) adults but 0% (0/13) children. Fever (temperature ≥38°C) at presentation was more frequent in children (38.5% [5/13]) than adults (4.7% [3/64]) (*P* < .005).

**Table 1.** Demographic, Clinical, and Laboratory Characteristics of the Patient Cohort, Adults and Children

	Median [Interquartile Range] or Count (%)		
	Adults (N = 64)	Children (N = 13)	*P* Value
Age at diagnosis, y	58.0 (40.0, 67.0)	12.3 (10.7, 13.8)	
Male sex	52 (81.3%)	8 (61.5%)	.146
Known prior tick exposure	15 (23.4%)	0 (0%)	.061
Known prior EM rash	2 (3.1%)	1 (7.7%)	.430
Fever	3 (4.7%)	5 (38.5%)	**<.005**
ESR (mm/h)	39 (24, 54)	38.5 (17, 51.5)	.860
CRP (mg/L)	53 (27, 99)	42 (13, 61)	.263
Synovial fluid WBC count (x 10^9^/L)	20 (12, 30)	53 (29, 65)	**<.005**
Synovial fluid %PMNs	84 (74, 90)	85 (77, 89)	.604
Serology testing	48 (75%)	13 (100%)	
IgG positive	47 (97.9%)	13 (100%)	
IgM positive	27 (56.3%)	7 (53.8%)	
Number of joints involved			
1	47 (73.4%)	12 (92.3%)	
2	13 (20.3%)	1 (7.7%)	
3	3 (4.7%)	0 (0%)	
4	1 (1.6%)	0 (0%)	
Knee involvement	63 (98.4%)	13 (100%)	1.000
Popliteal cyst	22 (34.4%)	3 (23.1%)	.529
Duration of symptoms prior to treatment, d	26 (11, 61)	8 (6, 34)	.132
Intra-articular steroids prior to treatment	12 (18.8%)	0 (0%)	.201

Abbreviations: EM, erythema migrans; ESR, erythrocyte sedimentation rate; CRP, C-reactive protein; WBC, white blood cell; PMN, polymorphonuclear leukocytes; IgG, immunoglobulin G; IgM, immunoglobulin M. Continuous variables are presented as medians with interquartile ranges and were compared using the Mann–Whitney *U* test. Categorical variables are presented as counts with percentages and were compared using Fisher's exact test. Statistically significant *P*-values are highlighted in bold.

Inflammatory markers were elevated in both groups. Synovial fluid analysis revealed that children had significantly higher synovial white blood cell (WBC) counts, with a median of 53 × 10^9^/L compared to 20 × 10^9^/L in adults (*P* < .005). Polymorphonuclear leukocyte (PMN) percentages in synovial fluid were similar between groups, with a median of 85% (IQR 77%–89%) in children and 84% (IQR 74%–90%) in adults. Serologic testing was performed in 48/64 (75%) adults and 100% (13/13) of children. IgG was positive in 97.9% of tested adults (47/48) and 100% (13/13) of children. IgM was positive in 56.3% of tested adults (27/48) and 53.8% of tested children (7/13).

Joint involvement patterns varied; monoarthritis was most common in both groups, present in 73.4% (47/64) of adults and 92.3% (12/13) of children. Oligoarthritis involving 2 joints occurred in 20.3% (13/64) of adults and 7.7% (1/13) of children. Three- and 4-joint involvement was rare and seen only in adults. Knee involvement was nearly universal, observed in 98.4% (63/64) of adults and 100% (13/13) of children. Popliteal cysts were identified in 34.4% (22/64) of adults and 23.1% (3/13) of children.

The median duration of symptoms prior to treatment initiation was longer in adults (26 days) compared to children (8 days), although this difference was not statistically significant (*P* = .132, Mann–Whitney *U* test). Intra-articular steroid administration prior to antibiotic therapy occurred in 18.8% (12/64) of adults and in none of the pediatric cases.

### Treatment and Response

**Figure 1.**
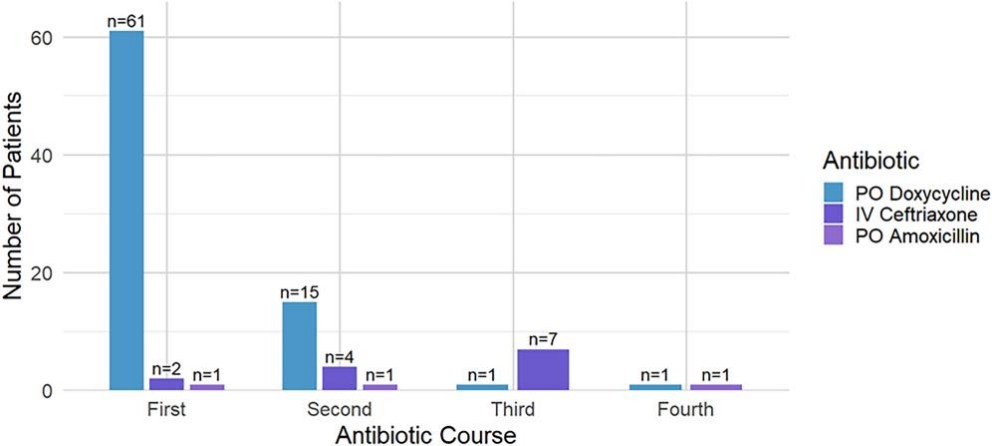
Antibiotic use across successive treatment courses in adult patients with Lyme arthritis. PO, oral; IV, intravenous. Figure created in RStudio [12].

**Figure 2.**
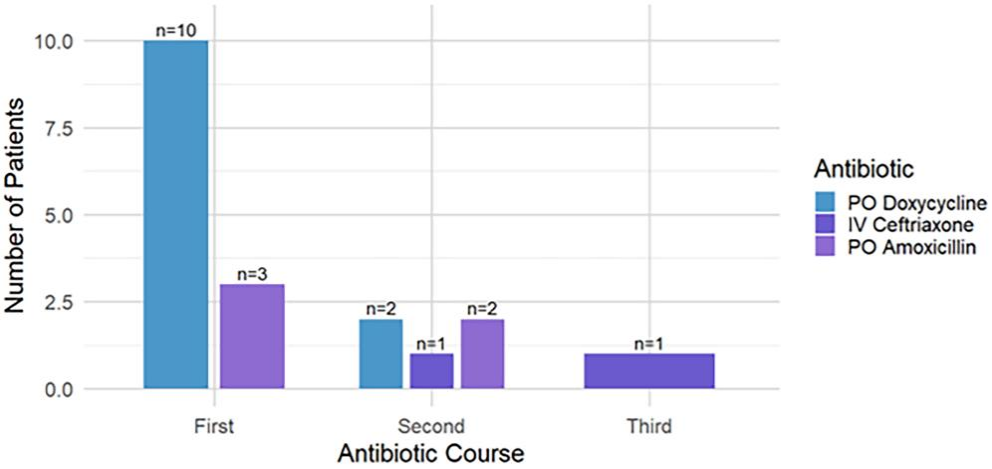
Antibiotic use across successive treatment courses in pediatric patients with Lyme arthritis. PO, oral; IV, intravenous. Figure created in RStudio [12].

Antibiotic prescribing patterns varied across successive treatment courses (Figure 1). Most adults were initially treated with oral doxycycline. The use of intravenous ceftriaxone in adults increased with subsequent courses. Among children, initial treatment mostly involved either oral doxycycline or amoxicillin, with intravenous ceftriaxone used later for a small number of cases (Figure 2).

**Figure 3.**
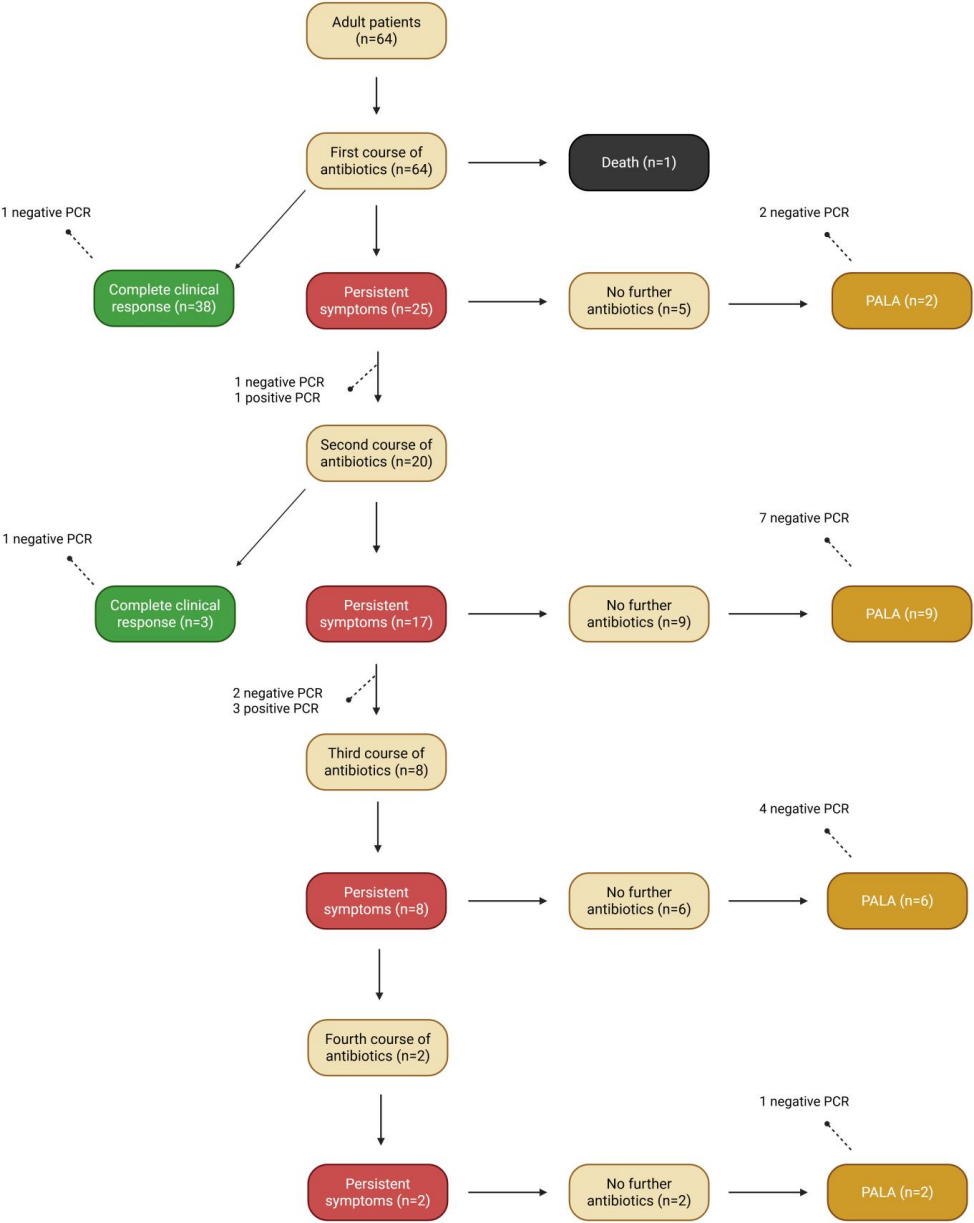
Sequential antibiotic treatment courses and outcomes in adult Lyme arthritis, including clinical response, persistent symptoms, polymerase chain reaction (PCR) results, and transition to postantibiotic Lyme arthritis. Among adult patients who underwent repeat synovial fluid PCR testing, the median time from initial PCR positivity to first documented PCR negativity was 89 d (IQR 71–133; range 21–207). Created in BioRender. Abu-Zeinah, K. (2026) https://BioRender.com/2w6vhl6.

Figure 3 demonstrates outcomes of treatment in the 64 adults. Following the first course of antibiotics, 59.4% (38/64) achieved complete resolution of symptoms. One patient died due to causes unrelated to Lyme disease. The remaining 25 had persistent symptoms, and most received additional antibiotic courses. After completion of antibiotic therapy, 64.1% (41/64) of adults achieved complete symptom resolution. Of the 23 adult patients who did not achieve symptom resolution after antibiotics, 78.3% (18/23) had microbiological response based on negative PCR testing. Ultimately, 29.7% of adults (19/64) progressed to PALA, including all patients who received 3 or more treatment courses. Seven PALA patients required disease-modifying anti-rheumatic drug (DMARD) therapy, and 3 required synovectomies. Notably, no patient receiving ≥3 antibiotic courses achieved complete symptom resolution, and all met criteria for PALA.

In the pediatric cohort, most patients (11 [84.6%]) had complete symptomatic resolution after antimicrobial therapy, 6 after the first course, 4 after the second, and 1 after the third. Of the remaining 2 patients with persistent symptoms, one had a negative PCR after the first course of antibiotics and was diagnosed with PALA, and the other had persistent intermittent symptoms after the first course but did not have repeat PCR testing or further antibiotics.

### Factors Affecting PALA Development

In our adult cohort, 19 patients progressed to PALA. Table 2 outlines the characteristics of the adult PALA cohort in comparison to the adult cohort that did not develop PALA. While the median age at diagnosis was lower in the PALA group (49.4 years [IQR 34.5–61.7]) compared to the non-PALA group (61.4 years [IQR 45.7–67.2]), this difference was not statistically significant (*P* = .24). The use of intra-articular steroids prior to antibiotic treatment was more common in PALA adults (31.6% [6/19]) than in those without PALA (13.3% [6/45]), but this difference did not reach statistical significance (*P* = .157). PALA adults also had a longer median duration of symptoms prior to treatment (47 days vs 17), but this was not statistically significant (*P* = .181). Notably, PALA adults had significantly lower synovial fluid white blood cell (WBC) counts (13.3 [IQR 9.7–22] × 10^9^/L vs 22.9 [IQR 14.2–32.3] × 10^9^/L, *P* = .014) and a higher median number of joints involved at initial presentation (1 [IQR 1–2] vs 1 [IQR 1–1], *P* = .011). No significant differences were observed between groups in synovial fluid PMN percentage, ESR, CRP, or presence of osteoarthritis (OA) on imaging. Adults without complete response to the first course of antibiotics were significantly more likely to develop PALA (0% [0/19] vs 84.4% [38/45], *P* < .005), and PALA adults were significantly more likely to have received IV antibiotics at any point during their treatment (57.9% [11/19] vs 6.7% [3/45], *P* < .005). Additionally, PALA adults received a significantly longer total duration of antibiotics during their treatment (56 days [IQR 56–84]) compared to adults without PALA (28 days [IQR 28–28]) (*P* < .005).

**Table 2.** Comparison of Clinical and Laboratory Features in Adult Patients With and Without PALA

	Median [Interquartile Range] or Count (%)		
	No PALA (N = 45)	PALA (N = 19)	*P* Value
Age at diagnosis, y	61.4 (45.7, 67.2)	49.4 (34.5, 61.7)	.240
Male sex	37 (82.2%)	15 (78.9%)	.739
Intra-articular steroids prior to treatment	6 (13.3%)	6 (31.6%)	.157
ESR (mm/h)	34 (21, 43)	62 (40, 81)	.075
CRP (mg/L)	61 (33, 113)	39 (22, 62)	.161
Synovial fluid WBC count (x 10^9^/L)	22.9 (14.2, 32.3)	13.3 (9.7, 22)	**.014**
Synovial fluid %PMNs	84.5 (77, 90)	79 (67, 88)	.190
Median number of joints involved	1 (1, 1)	1 (1, 2)	**.011**
Osteoarthritis on XR	18 (40%)	8 (42.1%)	1.000
Duration of symptoms prior to Treatment, d	17 (10, 40)	47 (12, 90)	.181
Complete response to first course of antibiotics	38 (84.4%)	0 (0%)	**<.005**
Use of intravenous antibiotics	3 (6.7%)	11 (57.9%)	**<.005**
Total duration of antibiotics, d	28 (28, 28)	56 (56, 84)	**<.005**

Abbreviations: EM, erythema migrans; ESR, erythrocyte sedimentation rate; CRP, C-reactive protein, WBC, white blood cell;, PMN, polymorphonuclear leukocytes; XR, X-ray. Continuous variables are presented as medians with interquartile ranges and were compared using the Mann–Whitney *U* test. Categorical variables are presented as counts with percentages and were compared using Fisher's exact test. Statistically significant p-values are highlighted in bold.

## DISCUSSION

In this retrospective study of PCR-confirmed native joint Lyme arthritis, we characterized clinical features and treatment outcomes of patients mostly residing in the Lyme-endemic regions of Minnesota and Wisconsin. These data reinforce several established aspects of Lyme arthritis and highlight clinically relevant nuances.

Consistent with prior epidemiological studies, we observed a male predominance in both adult and pediatric patients [6, 13, 14]. Most patients did not recall a history of tick exposure or an erythema migrans rash, highlighting the possible subtle presentation of Lyme arthritis and the need for a high index of suspicion in endemic areas. Lyme arthritis may be the first or only clinical manifestation of *B burgdorferi* infection in up to 30% of cases [8], likely because tick bites may be painless and easily overlooked. In addition, 20%–30% of infected individuals do not develop the classic erythema migrans rash [15]. Those who recognize a tick bite may receive prophylactic antibiotics, and patients with a rash usually receive early treatment; thus, our cohort likely represents individuals in whom the initial infection went unnoticed or untreated, allowing for disease progression and dissemination.

We noted differences in the presentation of Lyme arthritis between adults and children, although these findings should be interpreted cautiously given the small pediatric sample size. Compared to adults, children had significantly higher rates of fever and elevated synovial fluid white cell counts, along with a shorter, although not statistically significant, duration of symptoms prior to treatment compared to adults. These findings align with previous studies suggesting a more acute presentation in children but a more indolent course in adults [14]. The higher incidence of fever in children may reflect earlier presentation during the inflammatory phase or a more vigorous immune response [8, 16]. Knee monoarthritis was the most common presentation in adults and children, consistent with previous descriptions of Lyme arthritis phenotypes [8]. Popliteal cysts were also common, observed in approximately one-third of adults and one-quarter of children. These cysts can result from joint effusion and inflammation associated with Lyme arthritis [8], and in some cases, may be the initial manifestation of Lyme disease [17].

Among adults, 41 patients (64.1%) ultimately achieved complete symptomatic resolution following antibiotic therapy, 38 after the first course and 3 after the second. However, a significant proportion of adults (29.7%) developed PALA following antibiotic therapy, a rate slightly higher than previously reported figures of 10%–23% [2, 8, 9]. This higher observed rate may partly reflect cohort selection, as inclusion was restricted to synovial PCR-confirmed cases, which in practice are often obtained in patients with persistent symptoms or prolonged clinical course.

Notably, all adults who failed to improve symptomatically after 2 antibiotic courses, regardless of route, developed persistent inflammatory arthritis and were diagnosed with PALA. Failure to achieve symptom resolution after the first antibiotic course was associated with progression to PALA, suggesting that a lack of symptomatic response to initial antibiotic therapy, and most certainly the second, may predict those likely to develop chronic symptoms. Three adults with negative posttreatment synovial fluid PCR results were prescribed additional antibiotic courses; none had symptomatic improvement from prolonged therapy, and all progressed to PALA. Adults who developed PALA were more likely to receive intravenous antibiotics and longer total durations of therapy, likely reflecting clinician-directed treatment escalation in response to persistent or refractory symptoms. Prolonged antibiotic exposure did not appear to be associated with symptomatic resolution in these patients. These observations highlight that persistent symptoms following guideline-concordant therapy do not necessarily indicate ongoing infection and may not warrant further antibiotic therapy. Clinicians should consider alternative etiologies for persistent symptoms after appropriate antibiotic treatment, with PALA being an important consideration. Apart from PALA, other causes of persistent symptoms include post-Lyme disease syndrome myalgias/arthralgias, which may manifest as arthralgias without true synovitis, or osteoarthritis (OA) [8]. Radiographic evidence of OA was common among adults in our study, although it did not significantly differ between patients with complete versus incomplete symptomatic responses. Although there is a lack of studies particularly evaluating the effect of Lyme arthritis in accelerating OA, bacterial joint infections in general have been associated with accelerated osteoarthritic degeneration [18]. Thus, it is plausible that Lyme arthritis could similarly exacerbate joint degeneration.

In our cohort, PALA patients were more likely to have received steroids prior to diagnosis; however, this difference was not statistically significant. Previous studies examining the impact of preantibiotic steroid administration on Lyme arthritis outcomes have shown mixed results; some suggest an increased risk of persistent arthritis [19], while others have not found significant associations [8]. Steroids may blunt the immune response to *B burgdorferi*, potentially facilitating prolonged inflammation or persistence of antigenic debris. Delayed treatment with antibiotic therapy was associated with PALA, but this association was also not statistically significant. Our study may have been underpowered to detect significant effects of these variables on PALA development.

Adults who developed PALA in our cohort had significantly lower synovial fluid white cell counts prior to treatment, despite similar proportions of neutrophils compared to non-PALA patients. This observed association may have been confounded by factors such as pretreatment intra-articular corticosteroid use, duration of symptoms prior to aspiration, and extent of joint involvement. However, previous studies have indeed shown that PALA is characterized by dysregulated immune responses and distinct cytokine profiles that may impair leukocyte recruitment into the synovium early in infection [10, 20, 21]. Additionally, we observed more extensive initial joint involvement among patients who developed PALA, consistent with prior reports associating early multijoint involvement with an increased risk of persistent arthritis [9].

Our study has several limitations. Its retrospective design introduces potential biases inherent in relying on medical record documentation. As chart abstraction was performed by a single investigator, potential bias is also inherent in the study design. The small pediatric sample size restricts the generalizability of conclusions for this group, reducing our ability to draw meaningful conclusions regarding pediatric-adult differences in the presentation of Lyme arthritis. Restricting inclusion to patients with positive synovial fluid PCR likely limited the sample size and generalizability of our results. IDSA guidelines indicate serologic evidence and compatible clinical presentation alone are sufficient for diagnosis [8]; by restricting our cohort to those diagnosed via PCR, which has variable sensitivity, patients who otherwise met clinical and serological criteria were excluded. Additionally, since synovial PCR testing may, in practice, be performed in patients with persistent symptoms who undergo more extensive diagnostic evaluation via arthrocentesis, our inclusion criteria potentially enrich for more persistent presentations, possibly contributing to selection bias. Nevertheless, the stringent inclusion criterion of PCR positivity ensures stronger diagnostic specificity (Supplementary Appendix), reducing the likelihood of including patients in Lyme-endemic regions with positive serologies but joint symptoms from other etiologies. Although PALA classification required documentation of residual objective synovitis, misclassification bias remains possible given the retrospective design and reliance on clinical documentation. In particular, distinguishing PALA from osteoarthritis-related symptoms or other noninflammatory posttreatment musculoskeletal complaints may not have been possible in all cases. Finally, we were unable to precisely quantify time to symptom resolution or duration of PALA; follow-up assessments were symptom-driven, and those experiencing clinical improvement were less likely to attend subsequent evaluation. Transitions in care between specialties further limited precise temporal characterization.

Overall, our findings support a pragmatic clinical approach to the management of Lyme arthritis. In patients with objective inflammatory arthritis who fail to improve after an initial 4-week course of oral antibiotics, a second course of oral or intravenous therapy remains appropriate, in line with IDSA guidelines [8]. However, persistent arthritis despite 2 adequate antibiotic courses, particularly in the setting of negative repeat synovial fluid PCR or lack of symptomatic improvement with prior treatment escalation, should prompt consideration of PALA rather than ongoing infection. In such cases, further antibiotic therapy is unlikely to provide benefit and may contribute to unnecessary antimicrobial exposure. Repeat synovial fluid PCR testing may be considered selectively, but results should be interpreted cautiously given the potential for prolonged PCR positivity from nonviable bacterial DNA. Early referral to rheumatology is advisable for patients with suspected PALA for consideration of anti-inflammatory or DMARD therapy.

## CONCLUSION

This study highlights the clinical variability and treatment challenges of Lyme arthritis, particularly in patients who develop PALA. Our findings reinforce the importance of early recognition and treatment, but also caution against unnecessary antibiotic escalation in patients without symptomatic improvement. Persistent arthritis despite 2 antibiotic courses should prompt consideration of PALA and alternative etiologies rather than continued antimicrobial therapy.

## Supplementary Material

ofag368_Supplementary_Data

## Supplementary Data

Supplementary materials are available at *Open Forum Infectious Diseases* online. Consisting of data provided by the authors to benefit the reader, the posted materials are not copyedited and are the sole responsibility of the authors, so questions or comments should be addressed to the corresponding author.
