# Supplementary material for: Characteristics and Clinical Outcomes of Lyme Arthritis: A Retrospective Study
Source: Open Forum Infect Dis. 2026 Jun 19;13(6):ofag368. doi: 10.1093/ofid/ofag368 (PMC13293128; doi:10.1093/ofid/ofag368)
Supplement: ofag368_Supplementary_Data [file ofag368_supplementary_data.docx]

**Supplementary Table 1A:** Comparison of clinical consistency with Lyme arthritis among patients with ICD-coded Lyme arthritis and positive serology versus those with PCR-confirmed Lyme arthritis included in the primary analytic cohort. Clinical consistency was determined based on documentation of objective inflammatory arthritis and compatibility with a typical Lyme arthritis presentation. Approximately one-quarter of patients with positive Lyme serology did not have a clinical syndrome consistent with Lyme arthritis, highlighting that serologic positivity alone in a Lyme-endemic region carries a meaningful risk of misclassification

| **Characteristic** | **Serology-Positive Cohort* (N =113)** | **PCR-positive cohort (N=77)** |
| --- | --- | --- |
| Clinically consistent with Lyme arthritis | 86 (76%) | 77 (100%) |
| Not clinically consistent with Lyme arthritis | 27 (24%) | 0 (0%) |

* Positive Lyme serology was defined as positive IgG, with or without positive IgM, since Lyme arthritis is a late manifestation of Lyme disease, IgG positivity is expected (1).

PCR = Polymerase chain reaction

ICD = International Classification of Diseases

**Supplementary Table 1B:** Distribution of serologic and PCR test results among a subset of patients with ICD-coded Lyme arthritis in the designated study period who underwent both Lyme serologic testing and synovial fluid PCR testing at the time of diagnosis.

| **Group** | **Number (N = 67)** |
| --- | --- |
| Serology positive | 66/67 (98.5%) |
| PCR positive | 61/67 (91.0%) |
| Serology positive, PCR positive | 60/67 (89.6%) |
| Serology positive, PCR negative | 6/67 (9.0%) |
| Serology negative, PCR positive | 1/67 (1.5%) |

Appendix References:

1. Lantos PM, Rumbaugh J, Bockenstedt LK, Falck-Ytter YT, Aguero-Rosenfeld ME, Auwaerter PG, et al. Clinical Practice Guidelines by the Infectious Diseases Society of America (IDSA), American Academy of Neurology (AAN), and American College of Rheumatology (ACR): 2020 Guidelines for the Prevention, Diagnosis and Treatment of Lyme Disease. Clin Infect Dis. 2021;72(1):e1-e48. doi: 10.1093/cid/ciaa1215. PubMed PMID: 33417672.
